# ^1^H NMR-Based Metabolite Profiling of Plasma in a Rat Model of Chronic Kidney Disease

**DOI:** 10.1371/journal.pone.0085445

**Published:** 2014-01-20

**Authors:** Ju-Ae Kim, Hyo-Jung Choi, Yong-Kook Kwon, Do Hyun Ryu, Tae-Hwan Kwon, Geum-Sook Hwang

**Affiliations:** 1 Integrated Metabolomics Research Group, Seoul Center, Korea Basic Science Institute, Seoul, Korea; 2 Department of Chemistry, Sungkyunkwan University, Suwon, Korea; 3 Department of Biochemistry and Cell Biology, School of Medicine, Kyungpook National University, Taegu, Korea; 4 Graduate School of Analytical Science and Technology, Chungnam University, Daejeon, Korea; University of Florida, United States of America

## Abstract

Chronic kidney disease (CKD) is characterized by the gradual loss of the kidney function to excrete wastes and fluids from the blood. ^1^H NMR-based metabolomics was exploited to investigate the altered metabolic pattern in rats with CKD induced by surgical reduction of the renal mass (i.e., 5/6 nephrectomy (5/6 Nx)), particularly for identifying specific metabolic biomarkers associated with early of CKD. Plasma metabolite profiling was performed in CKD rats (at 4- or 8-weeks after 5/6 Nx) compared to sham-operated rats. Principle components analysis (PCA), partial least squares-discriminant analysis (PLS-DA) and orthogonal partial least squares-discriminant analysis (OPLS-DA) score plots showed a significant separation between the groups. The resulting metabolic profiles demonstrated significantly increased plasma levels of organic anions, including citrate, β-hydroxybutyrate, lactate, acetate, acetoacetate, and formate in CKD. Moreover, levels of alanine, glutamine, and glutamate were significantly higher. These changes were likely to be associated with complicated metabolic acidosis in CKD for counteracting systemic metabolic acidosis or increased protein catabolism from muscle. In contrast, levels of VLDL/LDL (CH_2_)_n_ and N-acetylglycoproteins were decreased. Taken together, the observed changes of plasma metabolite profiles in CKD rats provide insights into the disturbed metabolism in early phase of CKD, in particular for the altered metabolism of acid-base and/or amino acids.

## Introduction

Kidney is an organ which metabolizes a large number of substrates. Systemic metabolic disorder complicated in chronic kidney disease (CKD) is likely due to decreased renal function and altered metabolic activity of the kidney. These changes include disturbance of acid-base, water and electrolyte homeostasis, altered metabolism of glucose, amino acid, and lipid, accumulation of uremic toxins, and partial breakdown of endocrine function [1], [2], [3]. In particular, 3kidney plays a key role in the regulation of systemic acid–base balance by filtering blood and handling of acids and buffers. This includes the synthesis and secretion of ammonia, the excretion of titratable acids and free hydrogen ions, and the reabsorption and regeneration of bicarbonate (HCO_3_^–^) in the renal tubular epithelial cells [4].

In healthy individuals, systemic acid–base balance is maintained by the actions of both kidneys and lungs. When glomerular filtration rate (GFR) decreases in CKD, the balance is severely disturbed [5], [6], and metabolic acidosis could be complicated due to both decreased net acid excretion and impaired regeneration of bicarbonate [7]. In human patients, acid–base disorders caused by CKD are associated with a number of clinical manifestations, e.g., nausea and vomiting, electrolyte disturbances, increased susceptibility to cardiovascular events, activation of muscle proteolysis, and protein degradation [5], [8]. Moreover, animals with CKD induced by partial nephrectomy demonstrate that metabolic acidosis is associated with increased ammoniagenesis and activation of alternative complement pathway leading to tubulointerstitial inflammation and renal damage [9], [10]. Importantly, a recent study demonstrated that bicarbonate supplementation to correct metabolic acidosis in CKD patients slows the disease progression and improves nutritional status [11].

^1^H nuclear magnetic resonance (NMR) spectroscopy, a nondestructive chemical technique, provides detailed information on molecular structure, both for pure compounds and complex mixtures, as well as information on absolute or relative concentration of metabolites [12], [13]. The successful application of ^1^H NMR spectroscopy to plasma, urine, and other biofluids for studying altered metabolism in disease conditions has recently been established, and several important metabolites have been discovered as novel biomarkers for predicting the courses of diseases, such as diabetes mellitus or cardiovascular disease [13], [14], [15], [16], [17], [18]. In particular, we have recently demonstrated altered metabolic profiling in serum from human CKD patients with peritoneal dialysis or hemodialysis [12] and in the kidneys and urine from rats with lithium-induced nephrogenic diabetes insipidus [13]. Moreover, we did an integrated analysis of the transcriptome and metabolome in the kidney collecting duct cells, revealing that decreased extracellular osmolality is associated with decreased levels of organic osmolytes, glucose, intermediates of citric acid cycle, and branched chain amino acids [19]. In the present study, it is hypothesized that systemic metabolism, including metabolism of acid-base or amino acids, could be affected by renal failure and hence we aimed to identify specific metabolic biomarkers associated with early stage of CKD. The differences in the plasma levels of metabolites were investigated between rats with CKD induced by 5/6 nephrectomy (4- and 8-weeks) and corresponding sham-operated control rats by exploiting high resolution ^1^H NMR spectroscopy coupled with multivariate statistical analysis.

## Materials and Methods

### CKD animal model (4- and 8-weeks after 5/6 nephrectomy in rats)

Pathogen-free male Sprague-Dawley (SD) rats (180–200 g) were obtained from Charles River (Orient Bio, Seongnam, Korea). The animal protocols were approved by the Animal Care and Use Committee of the Kyungpook National University, Korea. Experimental CKD was induced by the excision of about two-thirds of right kidney and left total nephrectomy using the so-called excision remnant kidney model, as we previously demonstrated [20], [21]. Rats were anesthetized under enflurane inhalation. During surgery, they were placed on heated tables to maintain the rectal temperature at 37–38°C. Right kidney was exposed through right flank incision, gently dissected free from the adrenal gland, and approximately two-thirds of the right kidney, including the upper and lower poles, was excised. One week later, the rats were again anesthetized with enflurane inhalation, and left kidney was removed through left flank incision after dissecting it free from the adrenal gland. As a control group, rats were subjected to the sham operation identical to that used for the CKD rats except that their kidneys and poles were not removed. Both 5/6 nephrectomized CKD rats and sham-operated control rats were monitored for either 4- or 8-weeks.

### Plasma sample preparation

Plasma samples from the CKD rats and sham-operated rats at 4 weeks (CKD, n = 10; Sham, n = 10) or 8 weeks (CKD, n = 12; Sham, n = 12) were obtained after collecting venous blood from inferior vena cava in heparinized tubes (BD Vacutainer, Franklin Lakes, NJ). The plasma samples were stored at –80°C until NMR analysis. Before NMR analysis, the frozen plasma samples were thawed at room temperature and centrifuged (10 min at 13,000 rpm, 4°C). Next, 200 μL of the supernatant was mixed with 400 μL of saline buffer solution (10% D_2_O and 0.9% NaN_3_) in 5 mm NMR tubes for analysis [12], [15].

### ^1^H NMR spectroscopy

^1^H NMR spectra were acquired on a VNMRS 600-MHz NMR spectrometer at 25°C using a triple resonance 5 mm HCN salt-tolerant cold probe (Agilent Technologies Inc., Santa Clara, CA). The Carr-Purcell-Meiboom-Gill (CPMG) spin-echo pulse sequence (RD-90°-[τ-180°-τ] n-ACQ). A total T_2_ relaxation time of 60 ms was used to attenuate broad signals from proteins and lipoproteins. The ^1^H NMR spectrum was collected with a spectral width of 6720.4 Hz, relaxation delay of 2.0 s, and acquisition time of 4.0 s. Water solvent signal was suppressed by weak irradiating pulse on water peak during saturation delay. Free induction decay (FID) was acquired into 32,000 data points and the FID acquisitions were accumulated 128 times to increase signal-to-noise ratio. FIDs were weighted by an exponential function with a 0.3-Hz line broadening factor prior to Fourier transformation. All acquired NMR spectra were phase- and baseline-corrected, then referenced to the doublet at 1.32 ppm.

### Data processing of the NMR spectra and multivariate statistical analysis

All NMR spectra were phased and baseline-corrected using AMIX software (version 3.9.4; Bruker Biospin GmbH, Rheinstetten, Germany). Each ^1^H NMR spectrum from plasma collected 4 and 8 weeks after surgery was segmented into equal widths (0.005 ppm), corresponding to regions 0–9 ppm. The spectral data were normalized to the total spectral area. The data files were imported into MATLAB (R2008a; Mathworks, Natick, MA) and all spectra were aligned using the correlation-optimized warping method. The resulting data sets were then imported into SIMCA-P+ version 12.0 (Umetrics, Umea, Sweden), and all data were Pareto-scaled for multivariate statistical analysis. Principal components analysis (PCA), an unsupervised pattern recognition method, was performed to examine the intrinsic variation in the data set. To maximize the separation between samples, partial least-squares discriminant analysis (PLSDA), was applied. Permutation test was performed to check overfitting of the PLS-DA models. The PLS-DA models were validated using jackknife cross-validation [22]. Next, orthogonal partial least squares-discriminant analysis (OPLS-DA) was used to maximize covariance between the measured data (peak intensities from the NMR spectra) and the response variable (predictive classifications). The OPLS-DA color-coded coefficient loading plot was then used, which showed metabolites contributing to the separation between the CKD and sham-operated rats. The qualities of the models were described using R^2^ and Q^2^ values. R^2^, which indicates the goodness of fit, is defined as the proportion of variance in the data explained by the model. Q^2^, which indicates predictability, is defined as the proportion of variance in the data predictable by the model [14]. A non-overlapping ^1^H signal from each metabolite was used to calculate the integral area and the identified metabolites were quantified by relative peak intensity [23]. Resonant frequencies of each metabolite were referred from library of Chenomx NMR Sutie 7.1(Chenomx, Edomonton, Canada) and previous studies [12], [15], [24], [25], [26]. The Mann–Whitney test and Welch' t-test were performed to compare metabolite levels between the two groups. Statistical analyses were performed using Prism version 5 for Windows (GraphPad Software, San Diego, CA).

## Results

### CKD induced by 5/6 nephrectomy in rats

As shown in Table 1, CKD rats exhibited significantly decreased renal functions. The levels of plasma creatinine in CKD rats at 4 weeks (49.8±5.6 μmol/L, n = 10, *P*<0.05) and 8 weeks (59.7±3.9 μmol/L, n = 12, *P*<0.05) were significantly higher than their corresponding sham-operated control rats. Moreover, plasma urea nitrogen levels in CKD rats at 4 weeks (15.2±2.1 mmol/L, n = 10, *P*<0.05) and 8 weeks (16.2±1.2 mmol/L, n = 12, *P*<0.05) were significantly increased (Table 1). In addition, CKD rats had higher plasma osmolality and plasma potassium levels, whereas plasma sodium concentrations were unchanged (Table 1). The weight of remnant kidneys in CKD was significantly lower at 4 weeks (1.3±0.1 g, n = 10, *P*<0.05) than that of their corresponding sham-operated control rats (right kidney: 1. 6±0.1 g, n = 10, Table 1). In contrast, at 8 weeks the weight of remnant kidneys was significantly higher (2.2±0.2 g, n = 12, *P*<0.05) than their corresponding sham-operated control rats (right kidney: 1.6±0.1 g, n = 12, Table 1), indicating that the remnant kidneys in CKD were significantly hypertrophied.

**Table 1:** Changes of renal functions.

Parameter	Sham −4 wk	CKD −4 wk	Sham −8 wk	CKD −8 wk
n	10	10	12	12
BW (g)	435±8.2	390±7.9*	504±8.6	478±12.7
RK wt (g)	1.6±0.1	1.3±0.1*	1.6±0.1	2.2 ±0.2*
p-osm	299± 0.4	306±1.2*	297±0.7	307±1.5*
(mosm/KgH_2_O)				
p-Na^+^	136±0.4	135±0.9	136±0.2	136±0.3
(mmol/L)				
p-K^+^	3.8±0.1	4.2±0.1*	3.7±0.0	4.3±0.1*
(mmol/L)				
p- urea nitrogen	6.0±0.2	15.2±2.1*	5.7±0.1	16.2±1.2*
(mmol/L)				
p-Creat	19.4±1.2	49.8±5.6*	21.4±1.3	59.7±3.9*
(µmol/L)				

n, number of rats; BW, body weight; RK, right kidney in sham-operated control rats and remnant kidney in CKD rats; p-osm, plasma osmolality; p-Na, plasma sodium, p-K, plasma potassium, p-urea nitrogen, plasma urea nitrogen; p-Creat, plasma creatinine. *P<0.05.

### ^1^H NMR spectroscopic analysis of plasma

**Figure 1:**
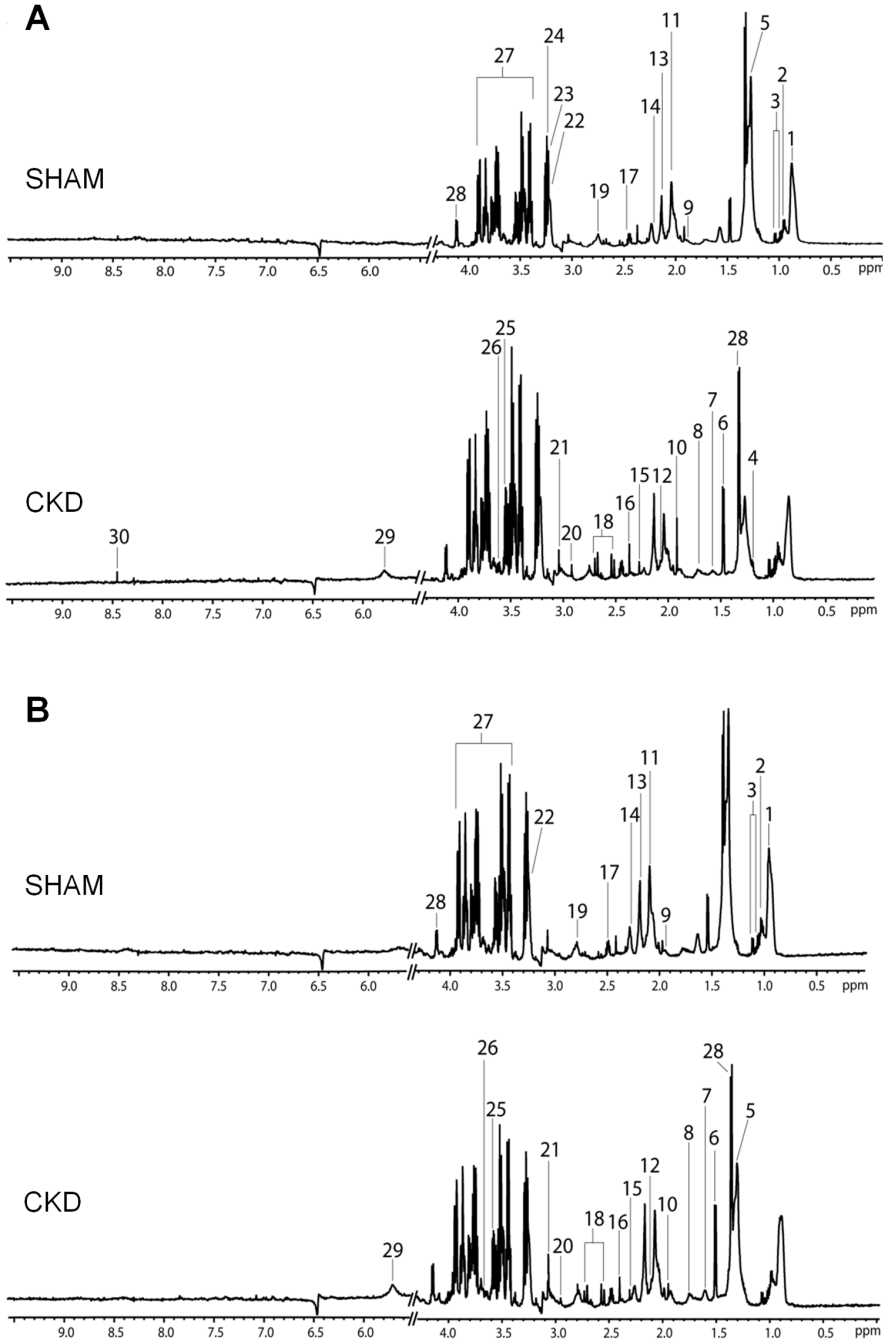
Representative 600 MHz ^1^H nuclear magnetic resonance (NMR) spectra of plasma obtained from 5/6 nephrectomized CKD rats and sham-operated control rats at 4 weeks (A) or 8 weeks (B) after 5/6 nephrectomy and sham operation. 1, VLDL/LDL CH_3_; 2, Leucine/Isoleucine; 3, Valine; 4, β-hydroxybutyrate; 5, VLDL/LDL (CH_2_)_n_; 6, Alanine; 7, Lipid CH_2_CH_2_C = O; 8, Lipid CH_2_CH_2_C = C; 9, Arginine; 10, Acetate; 11, N-acetylglycoproteins; 12, Glutamine; 13, Methionine; 14, Lipid CH_2_C = O; 15, Acetoacetate; 16, Pyruvate; 17, Glutamate/Glutamine; 18, Citrate; 19, Dimethylamine (DMA); 20, Trimethylamine (TMA); 21, Creatinine; 22, Choline; 23, Trimethylamine-N-oxide (TMAO); 24, O-phosphocholine; 25, Myo-inositol; 26, Glycine; 27, Glucose/Maltose; 28, Lactate; 29, Urea; and 30, Formate. CKD, chronic kidney disease.

The plasma metabolites were investigated using ^1^H NMR spectroscopy coupled with multivariate data analysis to identify the metabolic characteristics of early stage of CKD. ^1^H NMR (600 MHz) spectra of plasma samples obtained from sham-operated and CKD rats over 4 or 8 weeks are shown in Figure 1A and B. The spectral resonance of each metabolite was assigned on the basis of the 600-MHz library in Chenomx NMR suite 7.1 (Chenomx) and previous studies. Ambiguous peaks due to overlap or slight shifts were confirmed by spiking samples with the respective standard compounds. The plasma ^1^H NMR spectra contained a number of metabolites, including VLDL/LDL CH_3_, leucine, isoleucine, valine, β-hydroxybutyrate, VLDL/LDL (CH_2_)_n_, alanine, lipid CH_2_CH_2_C = O, lipid CH_2_CH_2_C = C, arginine, acetate, *N*-acetylglycoproteins, glutamine, methionine, lipid CH_2_C = O, acetoacetate, pyruvate, glutamate, citrate, dimethylamine (DMA), trimethylamine (TMA), creatinine, choline, TMA-N-oxide (TMAO), *O*-phosphocholine, myo-inositol, glycine, glucose, lactate, urea, and formate. Those metabolites showing the greatest difference between the CKD and sham-operated groups were VLDL/LDL CH_3_, β-hydroxybutyrate, VLDL/LDL (CH_2_)_n_, alanine, acetate, glutamine, methionine, acetoacetate, citrate, TMA, creatinine, lactate, and formate.

### Multivariate pattern recognition analysis

**Figure 2:**
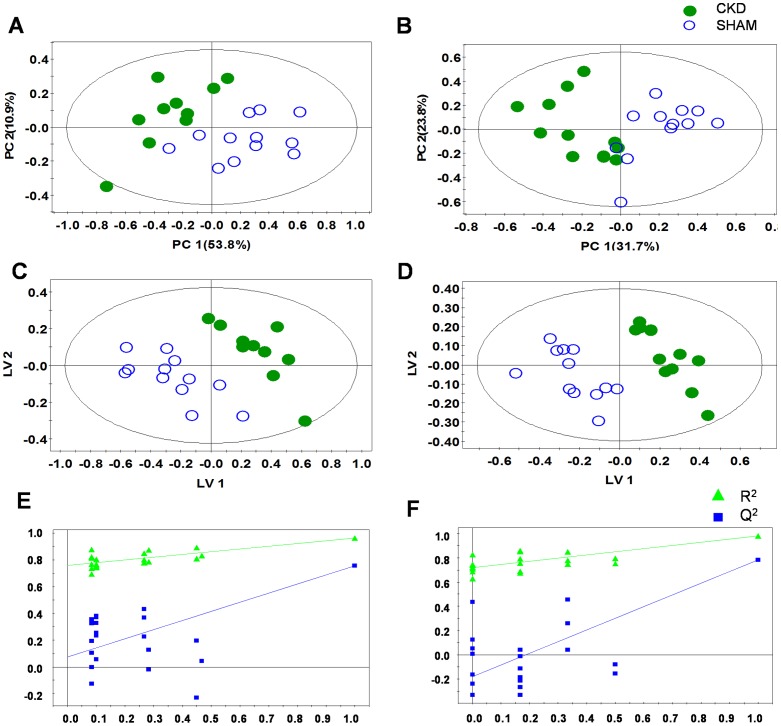
Principal component analysis (PCA), partial least squares-discriminant analysis (PLS-DA) score scatter plots and permutation tests of PLS-DA obtained from the ^1^H NMR spectra of plasma at 4 weeks (A, C, E) or 8 weeks (B, D, F) after 5/6 nephrectomy and sham operation, demonstrating a clear differentiation between the two groups. CKD, chronic kidney disease.

**Figure 3:**
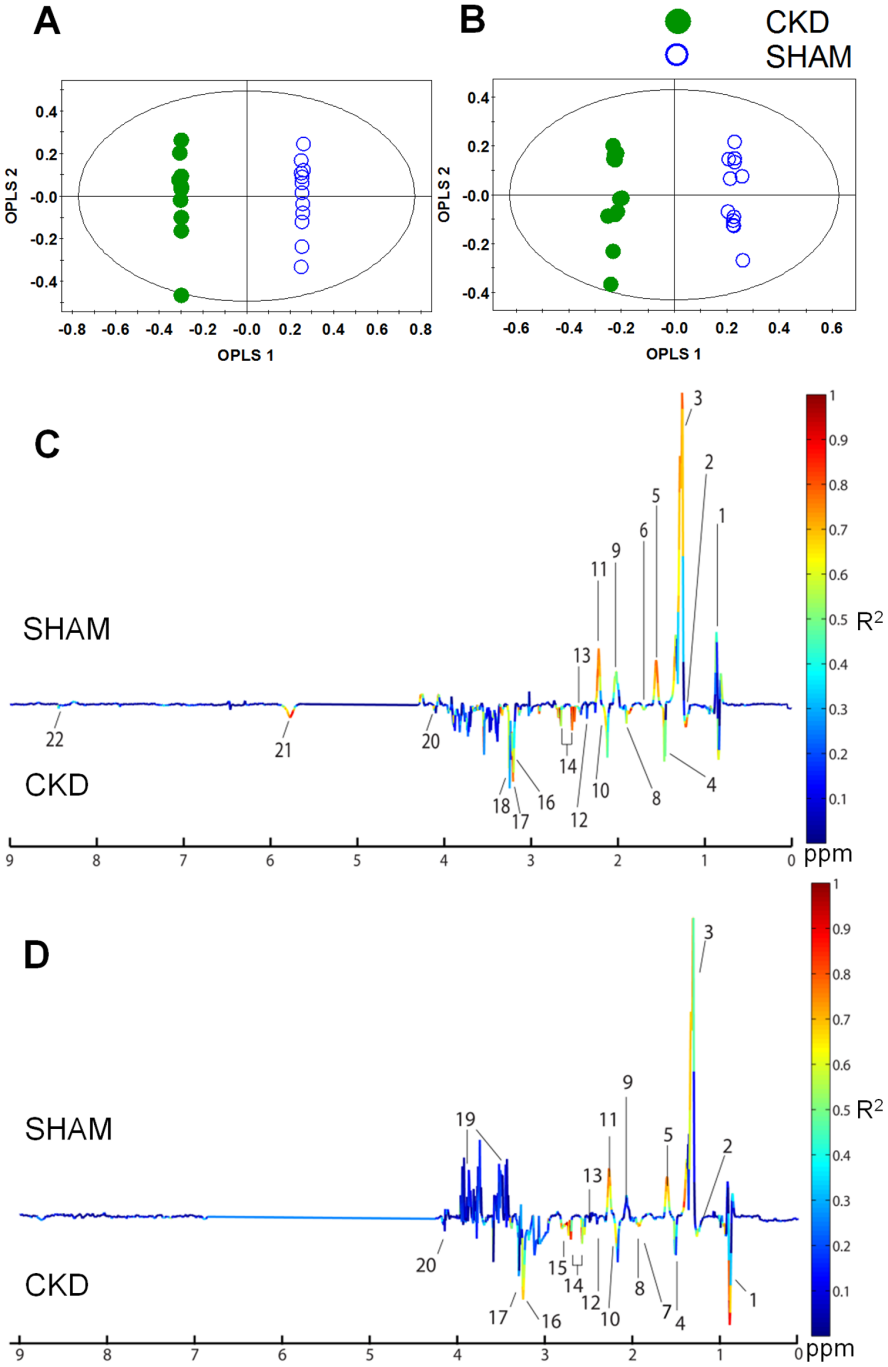
Orthogonal partial least-squares discriminant analysis (OPLS-DA) score and coefficient loading plots derived from the ^1^H NMR spectra derived from plasma at 4 weeks (A, C) or 8 weeks (B, D) after 5/6 nephrectomy and sham operation. The OPLS-DA coefficient loading plot shows a significant difference in the metabolite levels between the two groups. 1, VLDL/LDL CH_3_; 2, β-hydroxybutyrate; 3, VLDL/LDL (CH_2_)_n_; 4, Alanine; 5, Lipid CH_2_CH_2_C = O; 6, Lipid CH_2_CH_2_C = C; 7, Arginine; 8, Acetate; 9, N-acetylglycoproteins; 10, Methionine; 11, Lipid CH_2_C = O; 12, Acetoacetate; 13, Glutamine/Glutamate; 14, Citrate; 15, Trimethylamine (TMA); 16, Choline; 17, Trimethylamine-N-oxide (TMAO); 18, O-phosphocholine; 19, Glucose/Maltose; 20, Lactate; 21, Urea; and 22, Formate. CKD, chronic kidney disease.

To examine intrinsic variations between the two plasma data sets, PCA was initially performed using ^1^H NMR data [14]. As shown in Figure 2, the score plots for those samples collected at 4 weeks (Figure 2A: R^2^X = 0.722, Q^2^ = 0.562) and 8 weeks (Figure 2B: R^2^X = 0.672, Q^2^ = 0.493) were distinct between the CKD and sham-operated groups. Next, PLS-DA was employed, and it also indicated a clear difference, with increasing predictability of the separation between the two groups at 4 weeks (Figure 2C: R^2^X = 0.769, R^2^Y = 0.997, Q^2^ = 0.924) and 8 weeks (Figure 2D: R^2^X = 0.464, R^2^Y = 0.982, Q^2^ = 0.782). The PLS-DA models were validated using jackknife cross-validation. The accuracy of PLS-DA model for 4 weeks CKD models was 95% with 5 latent variables (LVs). The accuracy of PLS-DA models for 8 weeks CKD models was 100% with 3 LVs. The permutation tests of PLS-DA models showed that our models were not overfitted (Figures 2E and 2F). Next, to minimize the possible contribution of intergroup variability and to further improve the separation between the two groups, OPLS-DA was applied [15]. As shown in Figure 3, the score plots for 4 weeks (Figure 3A: R^2^X = 0.769, R^2^Y = 0.997, Q^2^ = 0.845) and 8 weeks (Figure 3B: R^2^X = 0.728, R^2^Y = 0.996, Q^2^ = 0.899) were clearly different between the CKD and sham-operated rats. The model parameters for explained variation, R^2^, and predictive capability, Q^2^, were calculated to assess the significance of the differences and were substantially high in plasma, indicating that the disease model of CKD was well established. Compared with the PCA model, our PLS-DA and OPLS-DA models indicated improved predictability, with increased Q^2^ values. In terms of monitoring time, although the value of Q^2^ did not exhibit a sharp difference, better separation between the two groups was seen at 4 weeks than at 8 weeks. An OPLS-DA coefficient plot, coded by color, was constructed to investigate the underlying variables contributing to the differentiation between the two groups. In the plot, negative peaks indicate higher metabolite levels in the plasma of the CKD rats compared to the sham-operated rats, while positive peaks indicate higher metabolite levels in the plasma of the sham-operated control rats.

As shown in the OPLS-DA coefficient loading plot (Figure 3), the CKD groups at 4 weeks (Figure 3C) and 8 weeks (Figure 3D) after 5/6 nephrectomy were characterized by elevated levels of β-hydroxybutyrate, lactate, alanine, acetate, acetoacetate, methionine, glutamine, glutamate, citrate, TMA, choline, phosphocholine, TMAO, urea, and formate, and by decreased levels of VLDL/LDL (CH_2_)_n_ and *N*-acetylglycoproteins, compared to the sham-operated control rats. In particular, the metabolites VLDL/LDL CH_3_, VLDL/LDL (CH_2_)_n_, lipid CH_2_CH_2_C = O, *N*-acetylglycoproteins, lipid CH_2_C = O, β-hydroxybutyrate, alanine, acetate, citrate, TMAO, and urea showed greater differences between the groups than the other metabolites, regardless of whether their levels were increased or decreased. These data indicate an altered metabolic pattern between the two groups, as well as the identities of the metabolites responsible for the observed differences.

### Quantitative analysis of the metabolites

**Figure 4:**
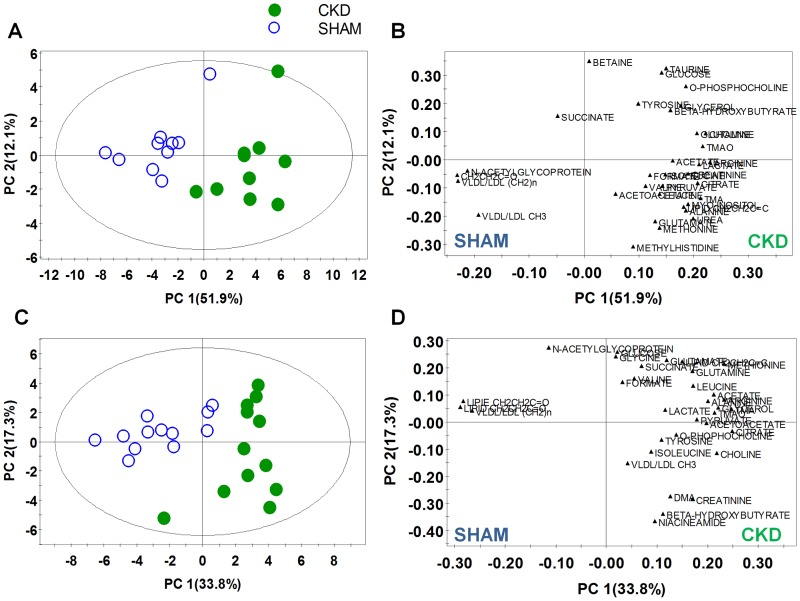
PCA score scatter and loading plots of metabolites quantification through targeted profiling of plasma at 4 weeks (A, B) and 8 (C, D), respectively. The loading plots were produced from the score plots which showed a significant differentiation between the two groups. CKD, chronic kidney disease.

**Figure 5:**
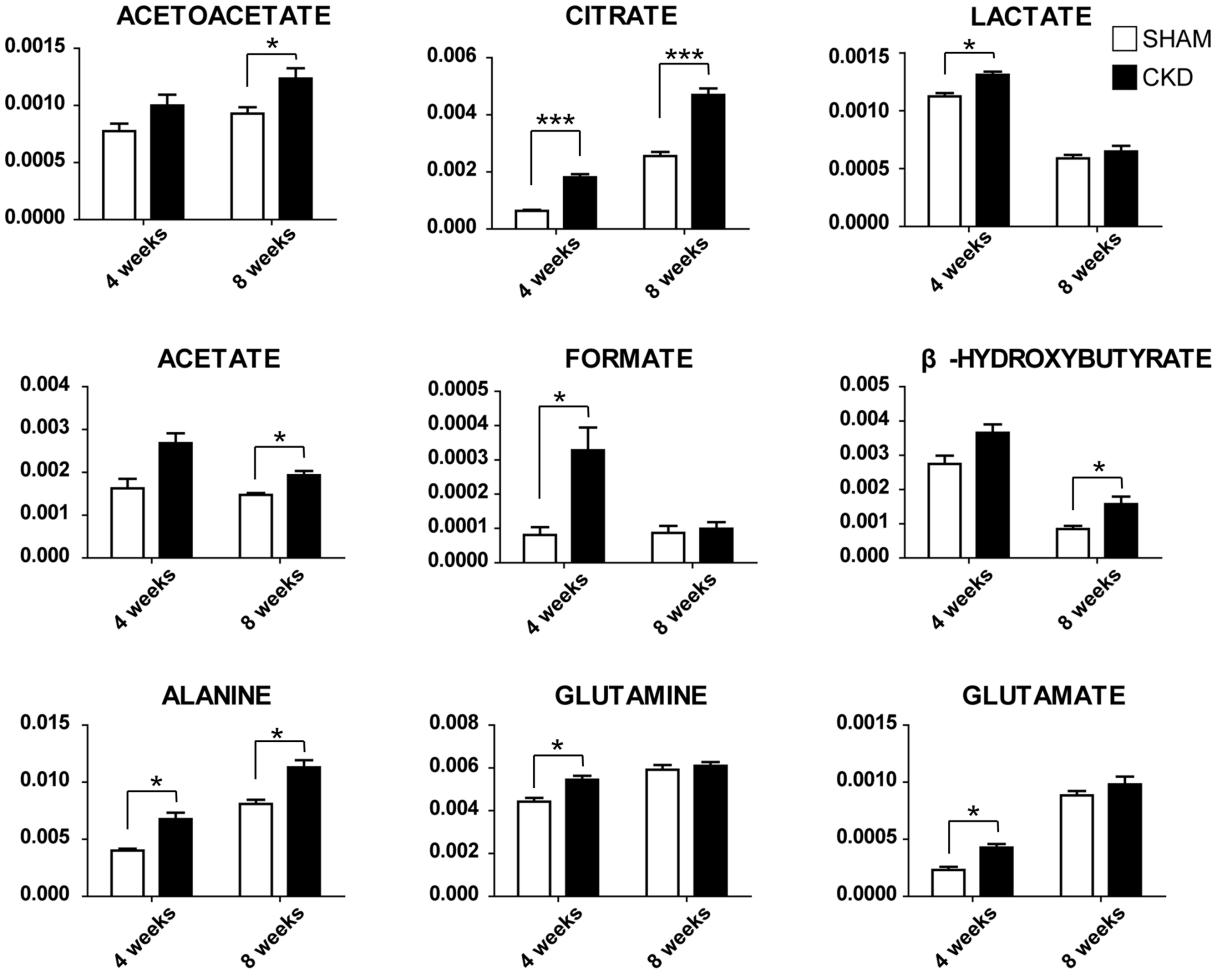
Quantification of changes of plasma metabolites in rats with CKD at 4 weeks and 8 weeks after 5/6 nephrectomy, respectively. *, **, *** indicate bonferroni corrected *P*<0.05, <0.01, and <0.001, respectively.

**Figure 6:**
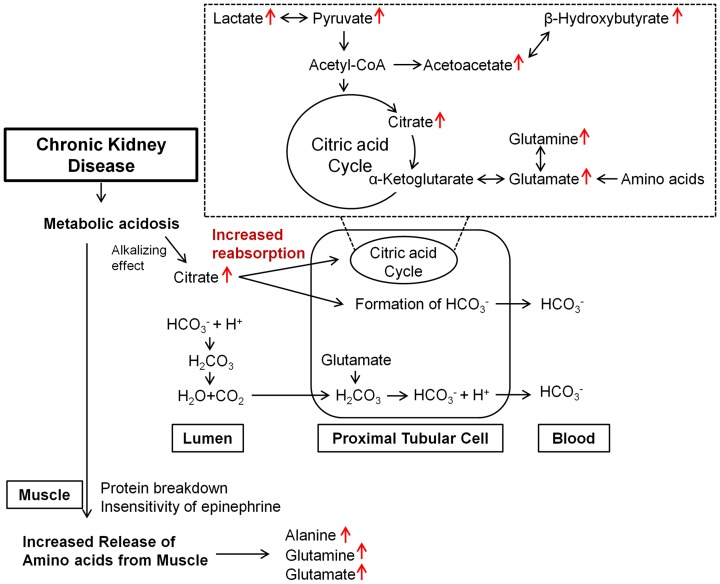
A diagram for the changes in plasma metabolites in CKD. The metabolite profiles of plasma in rats with CKD showed significantly increased levels of lactate, pyruvate, acetoacetate, β-hydroxybutyrate, glutamine, glutamate, and citrate. Metabolic acidosis is commonly complicated in CKD, due to both decreased net acid excretion and impaired regeneration of bicarbonate. This could change the citrate reabsorption in renal tubular cells, which metabolism generates HCO_3_^–^ ions producing an alkalinizing effect. Moreover, increased protein breakdown and insensitivity to epinephrine in muscle in CKD could induce the increases of alanine, glutamate, and glutamine in plasma.

NMR spectroscopy is a highly quantitative technique that can be used to accurately determine metabolite concentration, because integral area of metabolite peak is directly proportional to the metabolite concentration. A spectral peak from each metabolite was used to calculate the integral area and the identified metabolites were quantified by peak intensity. The metabolites were identified according to previous reports and the 600-MHz library from the Chenomx 7.1 NMR suite; several metabolites are displayed in Table 2. Differences in the levels of the relatively quantified plasma metabolites between the CKD and sham-operated groups were compared using multivariate analysis. PCA score plots derived from the quantification of plasma metabolites at 4 (Figure 4A; R^2^X = 0.72, Q^2^ = 0.39) and 8 weeks (Figure 4C; R^2^X = 0.723, Q^2^ = 0.279) show clear separation. In the same manner as global profiling, better separation of the two groups was shown at 4 weeks compared to 8 weeks based on the value of Q^2^. To identify the metabolites responsible for the differentiation between the two groups in the PCA score plots, PCA loading scatter plots were generated (Figure 4B, 4 weeks; Figure 4D, 8 weeks) through targeted profiling. Notably, on the basis of our quantitative data, most of the identified metabolites were substantially higher in the CKD rats than in the sham-operated rats. β-Hydroxybutyrate, lactate, alanine, acetate, acetoacetate, methionine, pyruvate, glutamine, glutamate, citrate, TMA, choline, *O*-phosphocholine, TMAO, creatinine, formate, and urea (4 weeks only) were elevated in the plasma of the CKD rats. In contrast, VLDL/LDL (CH_2_)_n_, lipid CH_2_CH_2_C = O, and *N*-acetylglycoproteins were decreased in the rats with CKD. These results are consistent with the above OPLS-DA color-coded loading plots (Figure 3C and D). Those metabolites that differed markedly between the CKD and sham-operated groups were β-hydroxybutyrate, alanine, lipid CH_2_CH_2_C = O, acetate, acetoacetate, methionine, glutamate, citrate, TMA, TMAO, urea (4 weeks only), and formate. To determine whether the differences between the CKD and sham-operated groups were significant, the Mann–Whitney test and Welch's t-test were performed after the Shapiro–Wilk normality test. The p-value from Mann-Witney test and Welch's t-test were adjusted by bonferroni correction for multiple comparisons. Those metabolites that were significantly different between the two groups are summarized in Table 2; the ratios of the relative metabolite concentrations in the CKD *vs.* the sham-operated group were shown in Table 2. In particular, significant increases in organic acids, including β-hydroxybutyrate, lactate, acetate, acetoacetate, citrate, and formate, and muscle-related amino acids were identified (Figure 5) and metabolic pathway related to the alteration of plasma metabolites in CKD was presented in Figure 6.

**Table 2:** ^1^H chemical shift and relative concentrations of metabolites observed in plasma from CKD and sham-operated groups.

Metabolite	Chemical chifts (δ 1H p.p.m)	Fold change ratio CKD/SHAM	
		4 weeks	8 weeks
VLDL/LDL CH_3_	0.36 (m), 0.86 (m)	0.853	1.077
Isoleucine	0.94 (t), 1.03 (d), 1.46 (m), 3.66 (d),	1.090	1.094
Leucine	0.94 (t), 0.99 (d), 1.70 (m), 3.72 (m)	1.156	1.076
Valine	1.00 (d), 10.3(d)	1.201	0.957
β-hydroxybutyrate	1.12 (d), 2.3 (dd), 2.4 (dd), 4.1 (m)	1.329	1.857 *
VLDL/LDL (CH_2_)_n_	1.25 (m)	0.55**	0.745*
Lactate	1.32(d), 4.13 (m)	1.164*	1.102
Alanine	1.48 (d), 3.81 (q)	1.689*	1.395*
Lipid CH_2_CH_2_C = O	1.56 (br)	1.296	1.124
Arginine	1.63–171 (m), 1.91 (m), 323 (t)	1.366**	1.471
Lipid CH_2_CH_2_C = C	1.7 (br)	0.335**	0.428**
Acetate	1.91 (s)	1.649	1.314*
N-acetylglycoproteins	2.04 (s)	0.844*	0.947
Glutamate	2.06 (m), 2.41 (m), 3.76 (m)	1.848*	1.109
Glutamine	2.12 (m), 2.44 (m), 3.76 (m)	1.231*	1.031
Methionine	2.15 (s), 2.16 (m), 2.65 (dd), 3.87(m)	1.953	1.199
Acetoacetate	2.26 (s), 3.49(s)	1.290	1.332*
Pyruvate	2.4 (s)	1.320	1.168
Dimethylanine	2.50 (s)	0.950	1.228*
Citrate	2.54 (d), 2.69 (d)	2.846***	1.834***
Trimethylamine	2.9 (s)	1.759***	1.379**
Creatinine	3.04 (s), 4.05 (s)	1.645	1.382
Choline	3.19 (s), 3.50 (m), 4.07 (m)	1.224**	1.104
Glucose	3.23 (t), 3.40–3.48 (m), 4.64 (d), 5.23 (d)	1.083	0.924
Trimethylamine-N-oxide	3.27 (s)	1.338*	1.148
O-phosphocholine	3.3 (s), 4.21(t), 3.61 (t)	1.157	1.034
Urea	5.8 (br)	3.748**	4 weeks only
Tyrosine	6.88(d),7.18(d)	1.220	1.213
Formate	8.45 (s)	4.049*	1.214

Letters in parentheses denote the peak multiplicities: s, singlet; d, double; t, triplet; dd, doublet of doublet; m, multiplets and br, broad; The fold change ratio indicates the ratio of relative concentrations between CKD and sham-operated groups; Statistical analysis was performed by Mann-Whitney test and Welch's t-test to assess the statistical significance between CKD vs. sham-operated groups; *, **, *** indicate bonferroni corrected *P*<0.05, <0.01, and <0.001, respectively.

## Discussion

We investigated the changes of plasma metabolites in CKD using ^1^H NMR-based metabolite profiling approach to understand the systemic disturbance of metabolism associated with the early stage of CKD. The metabolite profiles of plasma from rats with CKD showed significantly increased levels of organic acids, including citrate, β-hydroxybutyrate, lactate, acetate, acetoacetate, and formate, accompanied by increased alanine and glutamine levels.

In the present study, the levels of various metabolites in plasma were rapidly measured using the metabolite profiling procedure and thereafter the differences in the levels of plasma metabolites were compared using multivariate analysis, e.g., PCA-DA and OPLS-DA. The metabolic profiling and multivariate pattern recognition approach we have used herein allow us to observe simultaneously a wide range of metabolites which concentrations could be changed under the progression of CKD. Since metabolites can be regulated *via* a number of metabolic pathways, investigation of the whole feature of metabolites rather than several selected metabolites enables us to understand the underlying pathophysiological status of CKD more comprehensively. In this point, the current study differs from the previous reports and provides the significance of the observations.

In particular, plasma organic acids levels were significantly higher in rats with CKD. As the conjugate base of an acid, organic anions (e.g., citrate, β-hydroxybutyrate, lactate, acetate, acetoacetate, and formate) are potential bases, because they are readily metabolized HCO_3_^–^. Generally, renal contribution to acid–base homeostasis involves the regulated reabsorption of filtered HCO_3_^–^ ions and the regeneration of sufficient HCO_3_^–^ ions to neutralize the acid load. The kidneys also regulate alkaline compounds, including organic anions, to maintain the acid–base balance [4]. In the kidneys, HCO_3_^–^ is freely filtered in the glomerulus and the proximal tubule reabsorbs approximately 80% of the filtered HCO_3_^–^ and creates new HCO_3_^–^ by secreting H^+^ ions into its lumen through a Na^+^/H^+^ exchanger, and transporting HCO_3_^–^ ions *via* the basolateral Na^+^/HCO_3_^–^ co-transporter [27], [28], [29]. The secreted H^+^ ions react with filtered HCO_3_^–^ to form carbonic acid (H_2_CO_3_), which dissociates to CO_2_ and H_2_O. These substances then enter the cell, forming H_2_CO_3_. The H_2_CO_3_ is converted into H^+^ and HCO_3_^–^ intracellularly, thereby regenerating protons for the secretion into the lumen and the reabsorption of HCO_3_^–^ into the blood [4], [5], [30], [31], [32]. Next, the thick ascending limb of the loop of Henle reabsorbs 10–15% of the filtered HCO_3_^–^ that has escaped from the proximal tubule and then the distal tubule and collecting duct reabsorb nearly all of the remaining HCO_3_^–^ [4], [31], [33], [34], [35].

In CKD, reduced net acid excretion and/or impaired reabsorption and regeneration of HCO_3_^–^could result in systemic metabolic acidosis, due to the decreased glomerular filtration rates and the number of functioning nephrons. This was previously confirmed in CRF rats induced by 5/6 nephrectomy, demonstrating that blood pH and [HCO_3_^−^] levels were significantly decreased [36]. In this study, blood pH or [HCO_3_^−^] levels were not measured. However, CRF rats exhibited significantly increased blood urea nitrogen (BUN), creatinine, and potassium levels, compared with sham operated control rats. The finding indicated that renal function was significantly deteriorated and metabolic acidosis was likely to be associated with 5/6 nephrectomy.

Citrate is an important metabolic substrate in the kidneys and the most prevalent of the organic anions, accounting for about 10% of the energy production [37]. We demonstrated that citrate levels were significantly increased in plasma of rats with CKD. Citrate has three carboxyl groups, and consequently the metabolism of one citrate molecule generates three HCO_3_^–^ ions producing an alkalinizing effect [37], [38]. Therefore, retention of citrate and other organic anions by the kidneys is beneficial for counteracting metabolic acidosis, while increased excretion is beneficial for attenuating metabolic alkalosis [32]. Citrate is freely filtered by the glomerulus and up to 99% of the filtered load is reabsorbed in the proximal tubule *via* the apical membrane Na^+^/citrate co-transporter [38]. Metabolic acidosis increases renal citrate reabsorption and decreases urinary excretion. Consistent with this, Brennan *et al*. [39] demonstrated that decreasing the luminal pH caused an increase in citrate reabsorption, while increasing the luminal pH resulted in a fall in citrate reabsorption. During metabolic acidosis, therefore, the changes in blood pH could be minimized partly by the changes in renal citrate transport and metabolism, which are brought about by an adaptive increase in the activity of the Na^+^/citrate co-transporter [40]. The change of protein abundance of Na^+^/citrate co-transporter in the kidneys of rats with CKD needs to be determined.

We also demonstrated that rats with CKD exhibited elevated levels of alanine, glutamine, and glutamate. Alanine and glutamine are the prime precursors in hepatic and renal gluconeogenesis, respectively, accounting for two-thirds of the total amino acids released from the muscle [41], [42], [43]. Previous studies demonstrated that metabolic acidosis induced by CKD was associated with higher levels of alanine, glutamine, and glutamate in blood, possibly due to abnormal nitrogen utilization and accelerated net protein breakdown stimulated by muscle proteolysis [44], [45]. In addition, insensitivity to epinephrine affects the increases of alanine and glutamine in patients with CKD. Epinephrine is known to decrease the release and formation of alanine and glutamine by inhibiting the conversion of precursor amino acids to alanine and glutamine, and by reducing the rate of amino acid transport out of muscle cells [42]. Hence, the insensitivity to epinephrine observed in uremia, in turn, increases alanine and glutamine release from muscle [46].

In summary, we demonstrated the comprehensive systemic feature of metabolic disorders in the plasma in rats with CKD by exploiting ^1^H NMR-based metabolomic approaches combined with multivariate analyses. The metabolite profiles of plasma from rats with CKD demonstrated significantly increased levels of organic acids, alanine and glutamine levels. The observed changes of metabolites are likely to be clinically relevant to the well-known disturbed metabolism in early phase of CKD, in particular altered metabolism of acid-base and/or amino acids. However, since this study focused on 5/6 nephrectomy model, i.e. a surgical resection model of renal mass, further studies are needed to understand whether the observed changes in plasma metabolites are generally applicable to the CKD, irrespective of the underlying causes, e.g., diabetic kidney diseases or immune-mediated CKD.
